# Improving L2 learners’ IELTS task 2 writing: the role of model essays and noticing hypothesis

**DOI:** 10.1186/s40468-022-00206-0

**Published:** 2022-12-12

**Authors:** Long Quoc Nguyen, Ha Van Le

**Affiliations:** FPT University, Ho Chi Minh City, Vietnam

**Keywords:** IELTS, L2 learners, Model essays, Noticing, Output, Writing task 2

## Abstract

Achieving a sufficient IELTS band score for academic purposes has been a major goal of many L2 learners around the world, especially those in Asia. However, IELTS writing scores were consistently reported to be the lowest when compared to the scores in speaking, reading, and listening. Despite a growing body of research in IELTS writing, little focused on the role of model essays and noticing hypotheses. The present study aimed to fill in this gap by examining whether or not the implementation of both noticing hypothesis and model essays had a discernible influence on learners’ IELTS task 2 writing. To reach this goal, a quasi-experimental design including a pretest and a posttest was conducted with the voluntary participation of 52 undergraduates. These participants were divided into two groups: control group (CG, *n* = 25), learning in the conventional method (peer feedback and teacher feedback), and experimental group (EG, *n* = 27), using the noticing-model essays method. Following this, semi-structured interviews were performed to gain insights into the quantitative data. The results from this mixed-methods approach showed that there were significant gains in the overall performance and in the lexical resources subscale in the EG while no considerable changes were observed in the CG. Additionally, the other subscales (task response, grammatical range and accuracy, and cohesion-coherence) did not witness any significant differences between the two groups. Several pedagogical implications and recommendations for future research, especially in the Asian context, were also discussed.

## Introduction

Of all the four skills of English, writing has been deemed to be the most challenging for second language (L2) learners. This is evident via the international statistics of IELTS (International English Language Testing System), which demonstrated that the average band score in writing, an academic module, was the lowest when compared to that in the other three skills (Test taker performance 2021, n.d.). In particular, the scores for writing, speaking, reading, and listening in the year of 2021 were 5.92, 6.14, 6.26, and 6.50 (out of 9.0), respectively. To enhance L2 learners’ writing, corrective feedback (i.e., teacher feedback and peer feedback) has been widely researched and implemented (e.g., Hyland & Hyland, 2006; Allen and Mills, 2016; Yu et al., 2016; Hentasmaka & Cahyono, 2021). Effective as it has been shown, this kind of feedback does have several drawbacks. First, teachers’ or peers’ focus does not always match learners’ actual focus, which might lead to unfulfilled expectations (e.g., Izumi et al., 1999; Long & Robinson, 1998). Second, teacher feedback is not always available as teachers have to undertake a heavy workload, especially in large classes (Lee, 2003). Additionally, frequent and intensive exposure to input (feedback from teachers or peers) does not necessarily equate with native-like performances (Swain, 1985).

Consequently, there has been a shift in how L2 learners receive adequate feedback for their writing, which is to utilize output, noticing, and native speakers’ models. This technique has been demonstrated to be beneficial to learners’ L2 development (Eschholz, 1980; Smagorinsky, 1992a, 1992b; Lynch, 2009), and to be even “more helpful to the learner than error correction” (Qi & Lapkin, 2001, p. 286). Recent works have also confirmed the effectiveness of the noticing-model combination (Hanaoka, 2006, 2007; Hanaoka & Izumi, 2012; Khezrlou, 2021). In these studies, after learners composed their essays (output), they compared what they wrote with what native speakers did, identified the gaps or problems (noticing), and adopted the new information as input. Nonetheless, IELTS materials were not employed in such research, which warrants further exploration.

The method of using model essays together with noticing as a feedback instrument for learners’ IELTS writing has attracted increased, albeit still limited, interest from researchers (e.g., Bagheri & Zare, 2009; Qi & Lapkin, 2001; Tieu & Baker, 2022). These studies showed that learners who revised their essays based on this method achieved better band scores in writing compared to those receiving corrective feedback from teachers and peers. Prominent as deemed, there were still a few limitations in these works (i.e., only using qualitative data, unclear procedure, or low reliability) that rendered the results and findings questionable. Additionally, in the context of Asia, especially in Vietnam, except for Tieu and Baker’s study (2022), there seems to be an absence of sound research on the issue of noticing-model essays, which necessitates further investigation.

## Literature review

### IELTS academic writing

According to the book *IELTS Academic 17* (2022), the writing section consists of two tasks, the first one about describing a given diagram in at least 150 words (about 20 min) and the second one about composing an essay (advantages-disadvantages, opinions, causes-effects, causes-solutions, and both view discussions) in at least 250 words (about 40 min). While task 1 aims to evaluate test-takers’ ability to compare, contrast, organize and present data, task 2 focuses on their capability to respond to a given issue of various academic topics (IELTS Academic 17, 2022). The present study only concentrated on the second task as this part is about essay writing, and it weighs two times more than the first one.

In the public version of band descriptors, IELTS writing task 2 is marked based on four criteria, including task response (content), cohesion and coherence (unity and organization), grammatical range and accuracy (grammar), and lexical resources (vocabulary). The score is given on a scale of 9.0, with 0 being the lowest and 9.0 being the highest for each marking criterion. The overall grade is the average of the four subscores, rounded to .0 or .5. The detailed descriptions of the writing rubrics can be found in Appendix 1.

### Output, noticing, and language models in SLA

In second language acquisition (SLA), the output hypothesis was proposed and primarily discussed by Swain (1985, 1995, 1997, 2005), comprising three key elements: noticing, hypothesis testing, and metalinguistic awareness. The first function occurs when learners produce the target language from which they notice the problems preventing them from generating the intended meaning. This gap motivates learners to pay close attention to the required means of expressions that they need in order to convey the message successfully as intended. The second function is about learners’ trying out the information they have noticed earlier. Feedback is vital in this stage as they need to have something against which they can test their hypothesis (Swain, 1997). The last element is metalinguistic awareness which refers to learners’ reflection of the new information. They can do this by using it in contexts, which helps raise their awareness of the newly recognized language aspects.

There has been a considerable emphasis on learners’ attention and awareness as important elements (Swain, 1985; Schmidt & Frota, 1986; Swain and Lapkin, 1995; Schmidt, 2001). The credit for the noticing hypothesis was given to Schmidth (1983), who claimed that learners needed to be aware of their language use and test it against native speakers’ output. However, it was not until 2010 that he posited the official definition of this theory: “Input does not become intake for language learning unless it is noticed, that is, consciously registered” (p. 271). This definition highlighted the importance of noticing as the critical element for intake, which gained support from many linguists (e.g., Leow, 2018; Richards & Schmidt, 2013).

Negative input, also a kind of comprehensible input, was emphasized by Swain (1985), which linked input, noticing, and output together. As learners finished producing the language (output), they recognized (noticing) there were issues in their speaking or writing (gaps). Then, they compared their output with native speakers’ (negative input) and modified their language to concisely express their intended meanings (Rutherford & Smith, 1985; Swain, 1985). It can be seen that there is a general consensus among many researchers on the significance of the noticing hypothesis in SLA.

Although output, noticing, and native speakers’ models are vitally crucial to SLA, little research (Hanaoka, 2006, 2007; Hanaoka & Izumi, 2012; Khezrlou, 2021) has been conducted to explore their roles. In Hanaoka’s work (2006), which explored the effectiveness of native speakers’ models in enhancing noticing in L2 writing, the author employed a four-stage writing task including output, comparison, and two revisions with the participation of 37 Japanese learners at a women’s university. The participants were asked to write a narrative based on the given pictures and noted down any problems they had during their task (stage 1), compared their narratives with native speakers, and took notes on any linguistic features or differences they noticed (stage 2), rewrote their original text (stage 3), and rewrote it one more time after two months (stage 4). The results showed that models, as a feedback tool, played a significant role in promoting learners to notice the solutions to the problems they had and incorporate these features in their revisions. Using the same dataset, Hanaoka (2007) explored another aspect of output, noticing, and writing: learners’ attention to forms. The author reported that the participants overwhelmingly noticed lexical features (92.4%), found solutions to their problems, and implemented these elements in their revised versions.

Hanaoka and Izumi (2012) investigated how noticing and two feedback instruments (models and reformulations) helped solve learners’ overt and covert problems in L2 writing. The authors conducted the study via a multi-stage writing task with the participation of 38 Japanese EFL university freshmen (intermediate level of English). In the first stage, the students were required to write a story (a narrative paragraph of six sentences) based on picture prompts and take notes on any problems they had. In stage 2 (1 week later), they were given a model and a reformulated version of their writing (all written or modified by native speakers) and were asked to compare as well as note down any linguistic features or differences they noticed. In stage 3, they rewrote their narratives using the same pictures. It was found that the participants recognized solutions to both overt and covert problems and incorporated these new features in their rewritten paragraphs. Another finding was that while the models dealt with both overt and covert problems quite equally, the reformulations mostly addressed the overt issues.

The studies by Hanaoka (2006, 2007), as well as Hanaoka and Izumi (2012), highlighted the significant role of noticing and models in L2 writing, yet the findings seemed to be limited to revisions only. In other words, whether the same effect could be found when a new task is applied remains unknown. Khezrlou (2021) addressed this issue by exploring the effects of models between output of the same oral narrative task and the new task. Adopting a quasi-experimental design, the author divided 71 advanced beginner English as a foreign language (EFL) students into three groups: task repetition and oral modeling (group 1), task repetition and writing model (group 2), and task repetition with no modeling (group 3, control group). All groups took an additional oral narrative task 3 days later (after the treatment). The results demonstrated that group 1 and group 2 outperformed group 3 in terms of complexity, accuracy as well as fluency, and that group 2 (with writing modeling) performed better than group 1 (with oral modeling). It was also reported that while the number of error-free clauses remained unchanged, the number of accurate verb forms increased in task repetition with writing modeling and declined in the new task. Khezrlou (2021) concluded that models were effective in providing learners with linguistic features and also in expanding their language acquisition. However, Khezrlou’s research focused only on speaking; the effect of noticing and models on new writing tasks remains unanswered.

Overall, the extant literature on output, noticing, and models in SLA demonstrates that native-speakers’ modeling plays a significant role in promoting learners’ L2 development. Nonetheless, such literature is still limited, especially in writing. Although Hanaoka (2006, 2007), as well as Hanaoka and Izumi (2012), conducted studies on this skill, they neither used IELTS materials, which are far more complex than narratives, nor tested the effect of output, noticing, and models in a new task. These gaps necessitate further research.

### Empirical research on noticing-model essays in IELTS task 2 writing

Most studies about the combination of native speakers’ model essays and noticing in IELTS writing mainly employed descriptive analysis (Abe, 2009) or theme analysis (Baleghizadeh & Arab, 2011). Few researched this issue using experimental design, and even very few seemed to employ a mixed-methods approach to gain insightful data. In these studies, however, several limitations need to be addressed for higher validity and reliability.

Abe (2009) conducted an exploratory study on what language aspects Japanese L2 writers noticed when comparing their own essays with the model ones. After listening to the participants’ sharing (via speaking) and analyzing the frequencies of five categories (form, content, lexical, discourse, and others), the authors found that learners paid the most attention to lexical items. However, the sample size was only seven, and the participants’ noticing did not guarantee that they would write better. Therefore, it is still unclear whether the method of noticing-model essays is effective in improving learners’ L2 writing.

Bagheri and Zare (2009) explored the topic further, performing an experimental study with 65 Iranian university students divided into three groups: group A (intermediate learners, the baseline), Group B (intermediate learners, with model essays), group C (advanced learners, with model essays). After the treatment, the experimental groups (groups B and C) achieved higher scores in IELTS task 2 writing, outperforming the control group. However, Jafary (2014) conducted a similar study yet found that the experimental groups only did better in two aspects (task response and lexical resources). These two studies did not investigate whether there was a significant difference in each marking criterion of IELTS writing task 2 between the control and the experimental groups, only exploring learners’ perspectives on these aspects.

Recent research on noticing-model essays in IELTS task 2 writing in the context of Vietnam was performed by Tieu and Baker (2022). In a quasi-experimental design, they divided 33 undergraduates of intermediate level into two groups: the control group (*n* = 14) and the experimental group (*n* = 19). After the treatment, it was found that the experimental group who were exposed to model essays and noticing scored higher in all four aspects when compared to the control group. The posttest scores of the participants in the baseline group were even lower, albeit insignificant than their scores in the pretest. These results were, nevertheless, questionable because of three problems. First, the treatment only lasted 4 days, which seemed insufficient for such significant linguistic gains in the four criteria of IELTS task 2 writing. Second, the posttest was delayed for several months due to the COVID-19 pandemic, which might have altered the treatment in some ways as learners could have been exposed to other kinds of input or output. Third, the authors used *t* tests to compare the means, yet no normality tests were reported, raising doubts about the validity.

Consequently, further research is needed, especially with a mixed-methods approach, to alleviate the mixed findings and generate more reliable outcomes. This present study aims to fill in the aforementioned gaps by focusing on (a) whether model essays combined with noticing hypotheses improve learners’ overall scores in IELTS task 2 writing, (b) which of the four aspects (task response, lexical resources, grammatical range and accuracy, cohesion, and coherence) witnesses significant gains via the use of this feedback instrument. Two following research questions were formulated:

1. Does using noticing-model essays as a kind of feedback improve L2 learners’ overall scores in IELTS task 2 writing more than the conventional instructional method?
2. Does using noticing-model essays as a kind of feedback improve L2 learners’ scores in the four marking criteria of IELTS task 2 writing more than the conventional instructional method?

It is significant to conduct this research for several reasons. First, it contributes further to the literature on using model essays and noticing as a feedback instrument in improving learners’ output in IELTS task 2 writing, shedding light on the doubts in previous studies. Second, it helps ESL/EFL teachers and students, especially those in Asia, determine whether to employ this technique or not in IELTS preparation. In addition, it leads to a new path of research related to the incorporation of model output and noticing to enhance other skills of L2 learners.

## Methodology

### Participants

The participants in the present study were 52 EFL Vietnamese undergraduates aged 18–19, majoring in Software Engineering from two different classes. They took IELTS courses (listening-speaking and reading-writing) to achieve a required IELTS overall band score (6.0 out of 9.0) before officially enrolling in their specialized programs. They took the IELTS courses for 7 weeks, 6 days a week, from Monday to Saturday, 3 h a day. Every Monday, Wednesday, and Friday, they were trained the listening and speaking skills, while the other days were spent on reading and writing. Their English proficiency at the time of research, based on the placement test designed by Pearson Education (Ascher & Saslow, 2022), was B1 (CEFR: Common European Framework of Reference for Languages). The number of participants was very close to the required number yielded from the “a priori power analysis” test, which was 54, on the software G*Power 3.1 (Faul et al., 2009) with *α* = .05, power = .95, medium effect *f* = .25, ANOVA 2 × 2 within-between subjects design. Initially, 60 participants agreed to join the research, yet eight students’ data were discarded due to their absence from some of the writing sessions.

All of the participants took part in the research on a voluntary basis. They were clearly informed that their private data were kept strictly confidential and that their participation or withdrawal did not have any bearing on their official grades. Permission to conduct the study was also granted by the school’s management board.

### Research design

To address the research questions, a quasi-experimental design was adopted with a pretest and a posttest, followed by a semi-structured interview. Fifty-two participants were divided into two intact groups (also their classes). While the control group (*n* = 25, 18 males and 7 females) were trained in a conventional instructional method (with teacher feedback and peer feedback, no use of model essays), the experimental group (*n* = 27, 21 males, 6 females) learned writing via the use of model essays and noticing. All of them took the same pretest, different training, and the same posttest. After that, five students from the experimental group were randomly invited to join the interview for insightful data.

#### The pretest and posttest

In the pretest, all participants were given a writing question taken from the book* IELTS Academic 17* (2022) and were asked to write an essay of at least 250 words in 40 min in response to the question. They were not allowed to use any extra materials or to have any discussions during the test. After that, the two researchers, with years of IELTS training and certificates of “the IELTS Teacher Training Program 2020” granted by IDP Australia, marked the essays individually based on the IELTS task 2 writing rubrics (see Appendix 1), with a score (out of 9.0) given to each of the four criteria before the overall scores (rounded to .0 or .5) were calculated. Then, the two raters went through all the scores together and resolved all the discrepancies via discussions until both reached the final consensus.

In the posttest, the participants were required to write an essay of at least 250 words within 40 min on a given prompt. The question was extracted from the book “High-scoring IELTS Writing–Model Answers” (Fang & Wang, 2012); the theme was similar to the pretest question, yet the question was different. This was to ensure that the difference between the two tests was neither too large nor too small. The scoring process was the same as the one in the pretest, with separate markings before having discussions to reach the final agreement.

**Fig. 1:**
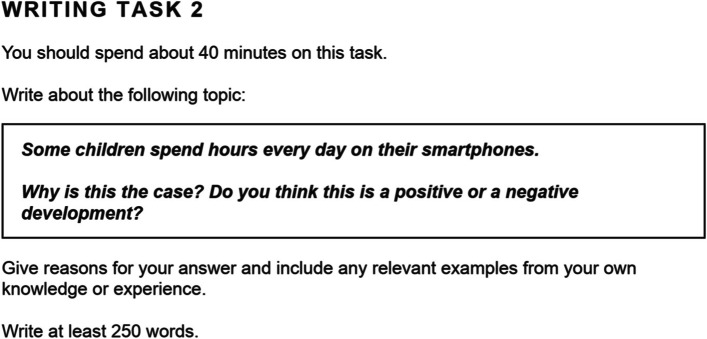
The pretest question

**Fig. 2:**
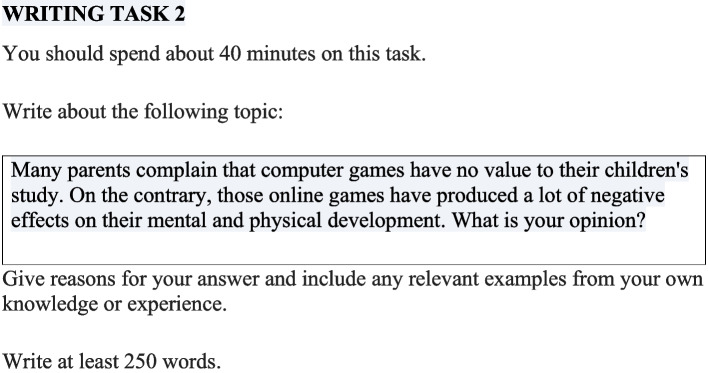
The posttest question

The following figures illustrated the questions in the two tests (Figs. 1 and 2).

#### The treatment

The control group received regular training using teacher feedback and peer feedback, which focused on any features of essays, such as grammar, vocabulary, ideas, and organization, to improve their writing, whereas the experimental group were exposed to the method of noticing-model essays. Both groups practiced writing on the same essay question taken from the e-book “The Key to IELTS Writing Task 2” by Cullen (2020), one of the authors of the Cambridge IELTS book series. This e-book not only gave formal and valuable instructions on IELTS essay writing but also provided readers with native-speakers’ model essays. The experimental group were given the model essays from this e-book as well as the ones from the book “High-scoring IELTS Writing–Model Answers” (Fang & Wang, 2012) to maximize their exposure to experts’ writing.

#### The interview

Five random participants from the experimental group were invited for the semi-structured interview, which was useful for making the interviewees feel at ease and sharing their ideas (Creswell & Creswell, 2017). There were six open-ended questions as fixed items on which follow-up questions could be based when necessary. Each interview lasted for about 5 min, and all were audio-recorded with the agreement of the participants. The language used was Vietnamese, the students’ L1, to avoid misunderstanding or ambiguity (Appendix 2).

### Procedure

The data collection stage took place within a 9-day time span. On day 1, all of the participants were given detailed information on the four criteria of IELTS task 2 writing and the rubrics (in Vietnamese to avoid misunderstanding) used to mark their writing before taking the pretest. On day 3 (days 2 and 4 were spent on the listening-speaking skills), they were asked to write an essay in response to a given question. Then, while the control group reviewed their friends’ essays and gave feedback before handing the papers with comments to their teacher for further evaluation, the experimental group were provided with two model essays from experts, taken from the e-book by Cullen (2020) and the book by Fang and Wang (2012), and asked to underline the parts they think were interesting or useful. Then, the students in the experimental group worked in pairs or groups of three and discussed with their partners what and why they thought were helpful in the model essays as well as how they could improve their original writing. During students’ discussions, the teacher went around the class and offered support to those with inquiries. On day 5, both groups were required to revise their essays based on the feedback (control group) or the model essays (experimental group). On day 8, all of the participants took the posttest and handed their papers to the teachers. On day 9, the researchers invited five students from the experimental group to join the semi-structured interview. The whole procedure could be summarized as follows (Table 1).

**Table 1:** Data collection procedure

Day	Control group	Experimental group
1	Received instructions on IELTS writing task 2—marking criteria and rubrics Took the pretest	
3	Worked in pairs and gave feedback Handed papers to the teacher for further evaluations	Received two model essays Underlined useful parts Worked in pairs and discussed what parts were useful, why they were useful, and how they could be implemented in the participants’ original essays Received support from the teacher, if necessary
5	Revised the original essays based on the feedback	Revised the original essays based on the model ones
8	Took the posttest	
9	Nothing	5 participants joined the interview after class

### Data analysis

The scores in the pretest and posttest were all analyzed in SPSS version 27 (Statistical Packages for Social Sciences). First, Shapiro-Wilk tests were run in order to examine the distribution of data; the results for pretest and posttest scores were presented in the following table (TR = task response, LR = lexical resources, GR = grammatical range and accuracy, CC = cohesion and coherence, OV = overall).

Tables 2 and 3 revealed that all of the scores in the pretest and posttest were not normally distributed (all the *p* values being under .01). Consequently, non-parametric tests were employed instead of *t* tests. Specifically, the Wilcoxon signed-rank tests were run to compare the participants’ performances between the pretest and posttest in each group, and the Mann-Whitney *U* tests were run to assess the results between the control group and the experimental group.

**Table 2:** Distribution of data of the pretest

Group	Criteria	***W***	***p***
Control (*n* = 25)	TR	0.805	< 0.01
	LR	0.714	< 0.01
	GR	0.762	< 0.01
	CC	0.786	< 0.01
	OV	0.861	< 0.01
Experimental (*n* = 27)	TR	0.742	< 0.01
	LR	0.801	< 0.01
	GR	0.789	< 0.01
	CC	0.701	< 0.01
	OV	0.785	< 0.01

**Table 3:** Distribution of data of the posttest

Group	Criteria	***W***	***p***
Control (*n* = 25)	TR	0.746	< 0.01
	LR	0.754	< 0.01
	GR	0.681	< 0.01
	CC	0.811	< 0.01
	OV	0.848	< 0.01
Experimental (*n* = 27)	TR	0.773	< 0.01
	LR	0.726	< 0.01
	GR	0.761	< 0.01
	CC	0.549	< 0.01
	OV	0.647	< 0.01

**Fig. 3:**
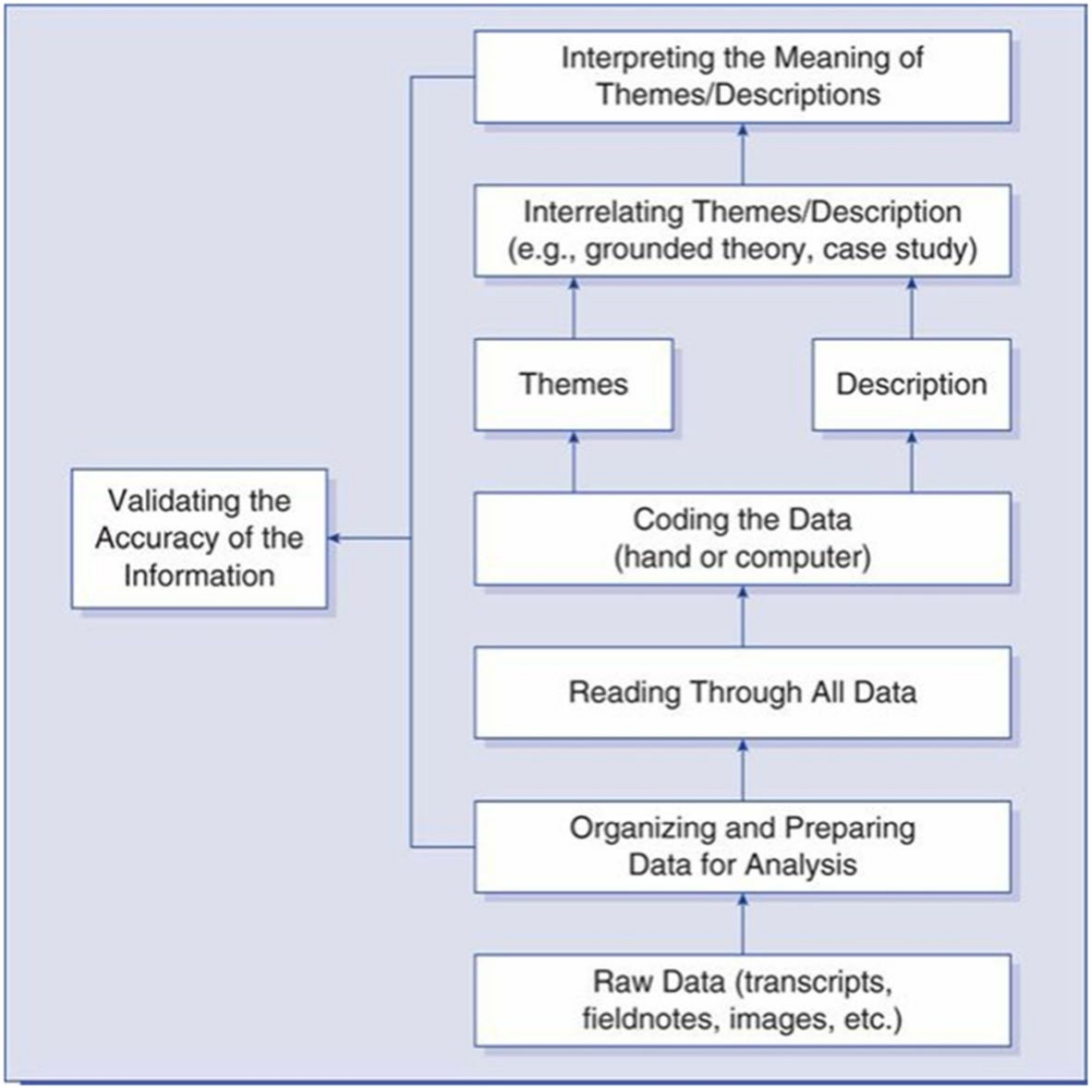
The 6-step strategy for qualitative data analysis by Creswell and Creswell (2017)

The interviews were analyzed based on the six-step strategy for qualitative data analysis proposed by Creswell and Creswell (2017). In particular, the researchers listened to the recordings carefully and transcribed verbatim. Afterwards, the researchers worked together to identify keywords, put them into codes, group the codes into categories, and combine them into two general themes. The results from the interview were used to explain quantitative outcomes from the non-parametric tests. The procedure is illustrated in the following model (Creswell & Creswell, 2017) (Fig. 3).

## Results

### Descriptive statistics

Tables 4 and 5 showed the descriptive statistics for the pretest and the posttest (values of Mean, SD, and 95% CI). It could be seen that the overall scores of the two groups ranged from 4.98 (B1, CEFR) to 5.41 (B2, CEFR) (IELTS in CEFR scale, n.d.). However, whether these changes were significant or not needed to be examined via the non-parametric tests.

**Table 4:** Descriptive statistics for the pretest

Group	Criteria	Mean	SD	95% CI
Control (*n* = 25)	TR	4.84	0.75	4.53–5.15
	LR	5.04	0.54	4.82–5.26
	GR	4.88	0.60	4.63–5.13
	CC	4.92	0.64	4.66–5.18
	OV	5.04	0.48	4.84–5.23
Experimental (*n* = 27)	TR	4.92	0.57	4.68–5.12
	LR	4.96	0.68	4.68–5.24
	GR	5.00	0.65	4.73–5.27
	CC	5.12	0.53	4.90–5.34
	OV	5.12	0.39	4.96–5.28

**Table 5:** Descriptive statistics for the posttest

Group	Criteria	Mean	SD	95% CI
Control (*n* = 25)	TR	4.89	0.58	4.66–5.11
	LR	4.96	0.59	4.73-5.20
	GR	4.89	0.51	4.69–5.09
	CC	5.07	0.73	4.79-5.36
	OV	4.98	0.43	4.81–5.15
Experimental (*n* = 27)	TR	4.81	0.62	4.57–5.06
	LR	5.41	0.56	5.18-5.63
	GR	5.33	0.62	5.09–5.58
	CC	5.26	0.45	5.08–5.44
	OV	5.41	0.24	5.31–5.50

Research question 1: Does using noticing-model essays as a kind of feedback improve L2 learners’ overall scores in IELTS task 2 writing more than the conventional instructional method?

**Fig. 4:**
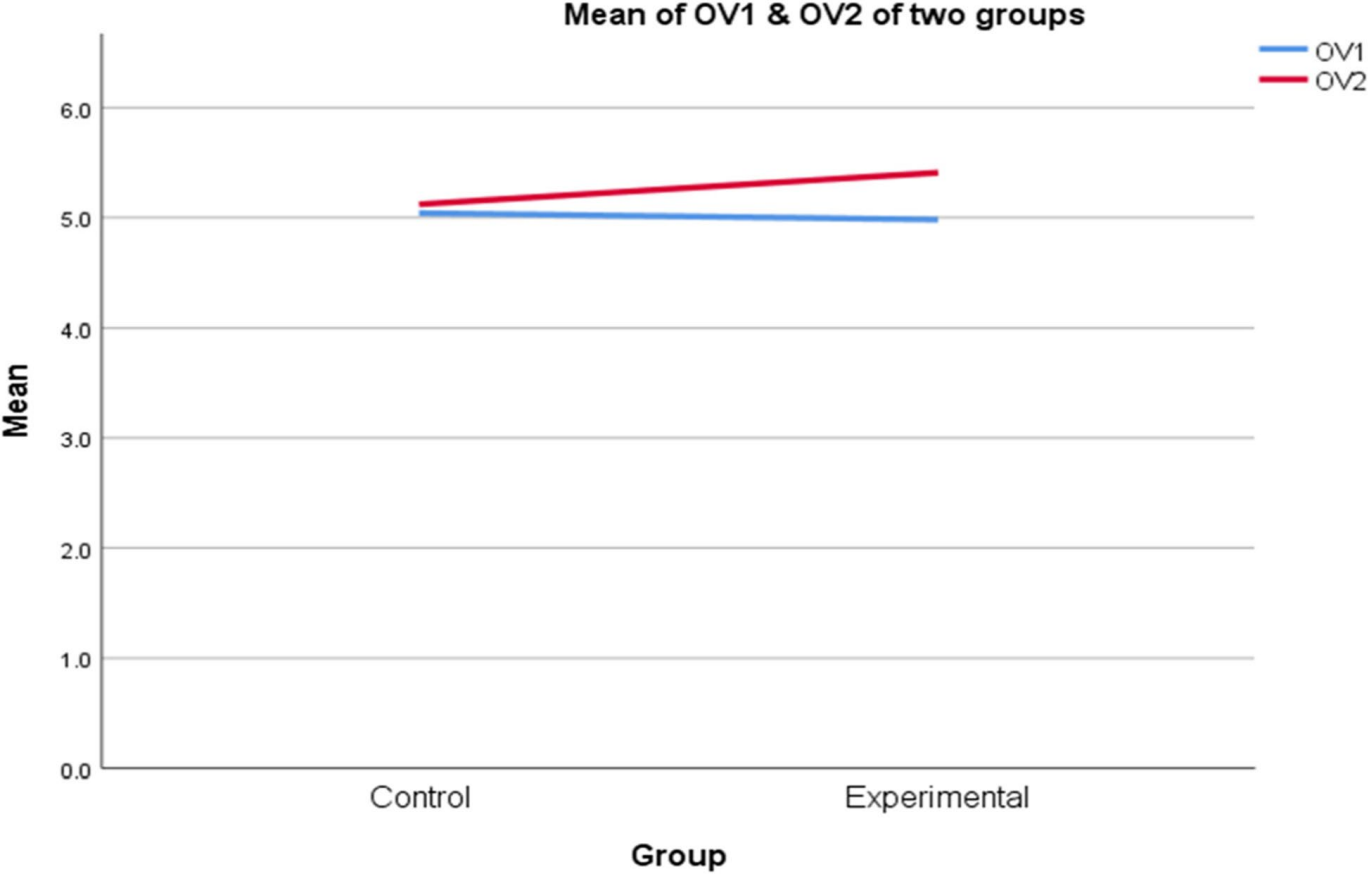
Comparisons of overall scores between two groups. OV1: overall scores in the pretest. OV2: overall scores in the posttest

As can be seen from Table 6, there was no significant difference in the pretest and posttest overall scores of the control group (*Z* = − .943, *p* = .346). In contrast, the experimental group achieved a significantly higher overall score in the posttest than in the pretest (mean difference = .29, *Z* = − 3.694, *p* < .001). As for the comparisons between the two groups, the Mann-Whitney *U* tests showed that although there was no significant difference in the overall scores in the pretest (*U* = 317.5, *p* = .699), the results in the posttest were significantly different (mean difference = − .43, *U* = 203, *p* < .001). Therefore, it could be concluded that using model essays with the noticing technique was more effective at improving L2 learners’ overall scores in IELTS task 2 writing than the conventional instructional method, which is demonstrated below (Fig. 4).

**Table 6:** Comparisons of the overall scores

	Mean difference	***Z/U***	***p***
Control group (CG) posttest–pretest	− 0.06	*Z* = − 0.943	0.346
The experimental group (EG) posttest–pretest	0.29	*Z* = − `3.694	< 0.001
Pretest: CG–EG	− 0.08	*U* = 317.5	0.699
Posttest: CG–EG	− 0.43	*U* = 203	0.005

Research question 2: Does using noticing-model essays as a kind of feedback improve L2 learners’ scores in the four marking criteria of IELTS task 2 writing more than the conventional instructional method?

#### Task response criterion

According to Table 7, it is obvious that there were no significant differences in the pretest and posttest scores of the task response criterion, either within or between the groups (all *p* values above .05). In other words, all of the participants were not able to improve their content-related aspects. It was also noticeable that those in the experimental group gained lower scores (mean difference = − .11), albeit insignificant, in the posttest than in the pretest.

**Table 7:** Comparisons of the task response scores

	Mean difference	***Z/U***	***p***
Control group (CG) posttest–pretest	0.05	*Z* = − 0.459	0.646
The experimental group (EG) posttest–pretest	− 0.11	*Z* = − 0.566	0.572
Pretest: CG–EG	− 0.08	*U* = 306.5	0.506
Posttest: CG–EG	0.08	*U* = 319.5	0.712

### Lexical resources criterion

Table 8 shows that despite the insignificant difference in the scores of lexical resources in the pretest between the two groups (*U* = 315.5, *p* = .620), the control group gained significantly lower outcomes than the experimental group in the posttest (mean difference = − .45, *U* = 220, *p* = .017). In addition, while the experimental group considerably improved their lexical use (mean difference = .45, *Z* = − 2.585, *p* = .01), the control group made no significant improvement (*Z* = 0.483, *p* = .629).

**Table 8:** Comparisons of the lexical resources scores

	Mean difference	***Z/U***	***p***
Control group (CG) posttest–pretest	0.04	*Z* = − .483	0.629
The experimental group (EG) posttest–pretest	0.45	*Z* = − 2.585	0.01
Pretest: CG–EG	0.08	*U* = 315.5	0.620
Posttest: CG–EG	− 0.45	*U* = 220	0.017

It could be inferred from Table 9 that learners in the experimental group significantly improved their grammatical use after the treatment (mean difference = .33, *Z* = − 2.585, *p* = .01), but those in the control group did not (mean difference = .01, *Z* = − .783, *p* = .434). However, when comparing the results in grammatical range and accuracy, the Mann-Whitney *U* tests revealed that there were no significant differences between the two groups, either in the pretest or the posttest, although the mean difference was greater in the posttest (− .44) than in the pretest (− .12).

**Table 9:** Comparisons of the grammatical range and accuracy scores

	Mean difference	***Z/U***	***p***
Control group (CG) posttest–pretest	0.01	*Z* = − .783	0.434
The experimental group (EG) posttest–pretest	0.33	*Z* = − 2.585	0.01
Pretest: CG–EG	− 0.12	*U* = 333	0.919
Posttest: CG–EG	− 0.44	*U* = 247.5	0.065

Similar to the results of the task response criterion, Table 10 demonstrates that there were no significant differences in the cohesion and coherence scores within each group and between the two groups (all the *p* values greater than .05). In other words, no considerable improvements were made in the two groups after the treatment.

**Table 10:** Comparisons of the cohesion and coherence scores

	Mean difference	***Z/U***	***p***
Control group (CG) posttest–pretest	0.15	*Z* = − 1.549	0.121
The experimental group (EG) posttest–pretest	0.14	*Z* = − 1.025	0.306
Pretest: CG–EG	− 0.2	*U* = 297.5	0.418
Posttest: CG–EG	− 0.19	*U* = 297.5	0.343

**Fig. 5:**
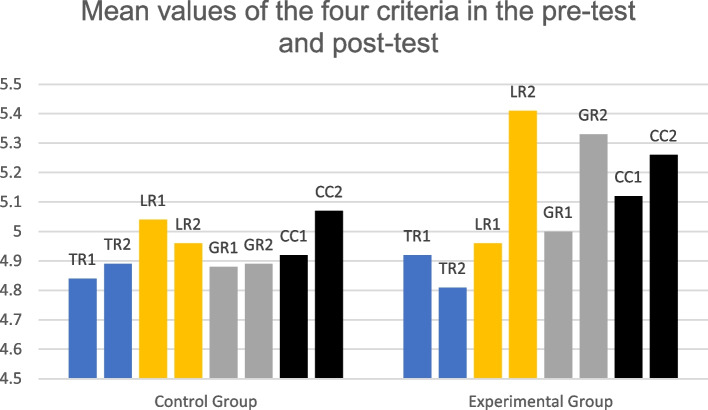
Comparisons of scores in the four criteria. TR = task response, LR = lexical resources. GR = grammatical and accuracy, CC = cohesion and coherence. 1 = pretest, 2 = posttest

In brief, through the detailed analyses, it is evident that using the noticing-model essays method significantly enhanced learners’ lexical resources while no considerable changes were observed in the task response and cohesion-coherence criteria. As for the grammatical range and accuracy aspect, although the treatment helped the participants somewhat improve their scores, this difference was statistically insignificant compared to the conventional method. A summary was demonstrated as follows (Fig. 5).

### Qualitative findings

After the interview data were analyzed, they were grouped into two general themes: positivity and negativity.

#### Positivity

When asked whether learning from model essays and noticing aided their IELTS task 2 writing, all of the interviewees (*n* = 5) agreed that they acquired a variety of useful expressions and terms that they previously did not know. They also tried to use the newly learned words in their posttest essays, which could be demonstrated via the sharings of participant 2 and participant 4:

“When I read the model essays, I realized that the authors and I had some similar ideas. But they expressed those ideas briefly yet still very correctly. I tried to memorize those expressions for my writing later.” (participant 2)

Agreeing with this viewpoint, participant 4 said, “I usually use repeated words in my essay, but after reading the model essays on the same topic, I knew some more synonyms and the ways to avoid repetition.” (participant 4)

Another positive feedback from the interviewees was that 80% (*n* = 4) favored this kind of learning as they were given the opportunity to practice writing in the way native speakers often did. Participant 3 highlighted this quite clearly, “In my previous class, the teacher gave a lot of feedback by circling all the mistakes and giving so many comments. It was too much for me to know how to fix all the mistakes. When I read the model essays, I know how to correct my mistakes because the answers are there, in the native-speakers’ essays.” Sharing the same view, Participant 5 said, “I like learning from the model essays because I can use them as tools to improve my writing, especially vocabulary.”

#### Negativity

Despite the favorable opinions, there were still several problems that the interviewees faced when using the noticing-model essays method. Sixty percent (*n* = 3) of the participants claimed that they did not have time to pay close attention to grammar, ideas, or organization because they focused too much on how to use the words from the model essays in their posttest. Participant 1 shared, “I was so eager to use the new vocabulary in my essay, so I spent a lot of time on this part. Then I had to rush to finish my writing. So I think I did not do well in other parts.”. Seeing eye to eye with participant 1, participant 4 said, “It is hard for me to focus on many things at the same time, vocabulary, grammar, ideas, and organization. So I think the model essays are good, but it takes time”.

There was one participant (20%) who did not really like this kind of learning because it required him to work too much. Details could be found in his sharing, “I can do this for once or twice, but not for a long time. I have to read and analyze too much. I feel tired”.

In brief, most participants held a positive attitude towards the use of native models’ essays as a feedback tool because they could learn useful expressions and terms which were incorporated in their revised and new-task essays. However, since they paid more attention to lexical items, other parts, such as grammar, organization, and ideas, were mostly overlooked.

## Discussion

Three major findings could be drawn from the analyses of the quantitative and qualitative data. First, it was found that via the implementation of model essays and the noticing hypothesis, learners’ overall scores in IELTS task 2 writing improved statistically significantly using the conventional instructional method. This finding aligned with previous research conducted by Tieu and Baker (2022) as well as Bagheri and Zare (2009). Such considerable improvements could be attributed to the features of the noticing hypothesis per se. Schmidth (1983) and Swain (1985) argued that being able to notice native-speakers’ model expressions helped learners identify their linguistic problems and try to acquire the model versions to fill in those knowledge gaps. This process eventually turned comprehensible input, specifically negative input, into intake.

Second, the method of using noticing and model essays significantly enhanced participants’ performances in the lexical resources criterion. This was in line with past studies which reported that learners used better and more accurate lexical items after being exposed to native speakers’ models (Bagheri & Zare, 2009; Jafary, 2014; Tieu & Baker, 2022). The explanation was that learners paid the most attention to this aspect of the model essays, as previously proved in the study by Abe (2009). Data from the semi-structured interview also showed that the participants in the experimental group spent a lot of time thinking about ways to incorporate newly learned words in their posttest. This was clearly explained by Schmidt and Frota (1986), claiming that learners who noticed the most differences between their original version and native speakers’ models would have the most gains.

The last finding was related to learners’ insignificant achievements in the other three criteria of IELTS task 2 writing, including task response, grammatical range and accuracy, and cohesion and coherence. This was in stark contrast to a recent empirical study (Tieu & Baker, 2022), which concluded that learners who used model essays and noticing gained significantly higher scores in these three aspects. The difference could be due to the fact that Tieu and Baker (2022) had to delay the posttest for three months under the impact of COVID-19. During that time, the participants might have been exposed to other factors, leading to significant achievements in all areas of IELTS task 2 writing. Another plausible explanation was that in this present study, as in the semi-structured interview and also in the study of Hanaoka (2007), the participants focused too much on lexical items, thereby deterring them from having sufficient time to spend on other elements like grammar, organization, and ideas. In other words, lexical resources attracted most of their attention, so little was left for the other criteria.

Several pedagogical implications can be drawn from the present study. First, teachers and institutional leaders should make a detailed plan to implement model essays in the writing training curriculum, especially IELTS task 2 writing, due to its significant benefits to learners’ achievements. As argued by Muranoi (2007), “providing learners with opportunities for producing output in language-use contexts (i.e., language models) is facilitative in developing learners’ interlanguage, especially productive skills” (pp. 76–77). Second, to maximize students’ attention, one writing session should focus on only one or two linguistic aspects (i.e., grammar, vocabulary, ideas, and organization). This is because students’ concentration and noticing are still limited, and they should practice this kind of learning gradually until they are more familiar. Finally, evaluations or feedback (either from peers or teachers) are vital to the noticing-model essays method as students need to think aloud (express) their noticing to their friends or teachers who give them confirmation on whether their use of newly learned expressions (testing hypothesis) is appropriate. In other words, teachers are recommended to offer students timely support to reinforce the acquisition obtained from model essays.

## Conclusion

The aim of the present study was to investigate whether the combination of the noticing hypothesis and model essays made any significant difference in learners’ IELTS task 2 writing. Three major findings were generated via the employment of a mixed-methods approach, including a quasi-experiment and semi-structured interviews. First, the noticing-model essays method significantly enhanced learners’ overall scores in IELTS task 2 writing, while the conventional instructional method did not have any considerable effect on learners’ overall scores. Second, after the treatment, a participant in the experimental group gained significantly higher scores in the lexical resources criterion. Third, the other three aspects (task response, grammatical range and accuracy, and cohesion-coherence) did not witness any statistically significant changes. Several pedagogical implications were provided, including the call for the implementation of this method, the focus on only one or two linguistic areas in each writing session, and appropriate feedback or support from peers and teachers. However, there are a number of limitations that should be addressed. For one thing, as the present study only examined learners’ exposure to model essays for a short time (within 9 days), future research is recommended to measure the effect of the method for a longer time. Besides that, the theme of the question in the posttest was still similar to that in the pretest, so whether similar outcomes can be achieved if the theme is entirely different remains unknown. In addition, as learners are more likely to focus on lexical items, which lessens the effect on other writing criteria such as task response, grammatical range and accuracy, and cohesion-coherence, future studies could specifically draw students’ attention to only one or two criteria at a time to examine whether significant improvements can be made.

## Figures and Tables

**Fig. 6:**
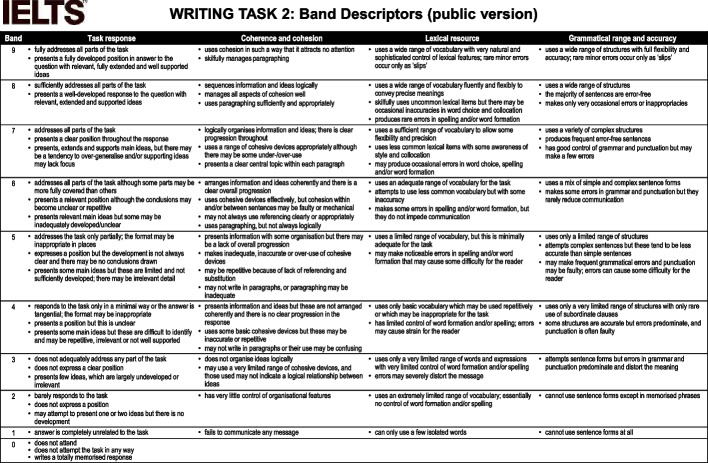
Writing rubrics

## Data Availability

All the data used in this study belong to the authors and will be shared upon reasonable request.

## Appendix 1

Fig. 6

### Appendix 2

Interview questions

1. Do you think model essays can help you improve your vocabulary in IETLS Task 2 Writing? Please explain.
2. Do you think model essays can help you improve your grammar in IETLS Task 2 Writing? Please explain.
3. Do you think model essays can help you organize ideas in IETLS Task 2 Writing better? Please explain.
4. Do you think model essays can help you get better ideas for IETLS Task 2 Writing? Please explain.
5. What difficulties do you face when using model essays in your writing class? Please explain.
6. Do you like learning writing through model essays? Please explain.

## Abbreviations

CC: Cohesion and coherence
CEFR: Common European Framework of Reference for Languages
CG: Control group
EFL: English as foreign language
EG: Experimental group
ESL: English as second language
GR: Grammatical range and accuracy
IELTS: International English Language Testing System
L2: Second language
LR: Lexical resources
OV: Overall score
SLA: Second language acquisition
SPSS: Statistical Packages for Social Sciences
TR: Task response
